# Buspirone Induces Weight Loss and Normalization of Blood Pressure via the Stimulation of PPAR*δ* Dependent Energy Producing Pathway in Spontaneously Hypertensive Rats

**DOI:** 10.1155/2023/7550164

**Published:** 2023-04-30

**Authors:** Yong-Jik Lee, Hyun-Min Kim, Yoo-Na Jang, Yoon-Mi Han, Hong Seog Seo, Tae Woo Jung, Ji Hoon Jeong, Hyun Jung Lee, Kyung Oh Jung

**Affiliations:** ^1^Cardiovascular Center, Korea University Guro Hospital, 148, Gurodong-ro, Guro-gu, Seoul 08308, Republic of Korea; ^2^Department of Anatomy, College of Medicine, Chung-Ang University, Seoul, Republic of Korea; ^3^Department of Medical Science, BK21 Plus KUMS Graduate Program, Korea University College of Medicine, Seoul, Republic of Korea; ^4^Department of Medicine, College of Medicine, Graduate School, Chung-Ang University, Seoul, Republic of Korea; ^5^Department of Pharmacology, Chung-Ang University College of Medicine, Seoul, Republic of Korea; ^6^Department of Global Innovative Drugs, Graduate School of Chung-Ang University, Seoul, Republic of Korea

## Abstract

**Introduction:**

Buspirone, as a partial agonist for a 5-hydroxytryptamine (serotonin) receptor 1A (5-HT1A), has been prescribed as an anxiolytic drug for patients. In addition, the lowering effect of serotonin on blood pressure was reported in hypertensive animal model. We investigated the therapeutic mechanism of buspirone against lipid metabolism disturbed by hypertension of early stage via hypertensive and obese animal model.

**Methods:**

The levels of various biomarkers related to lipid metabolism and hypertension were estimated through the measurement of body weight and fat weight, blood analysis, western blotting, immunohistochemistry, and staining methods.

**Results:**

The lipid accumulation was lowered in differentiated 3T3-L1 cells by buspirone treatments of 50 and 100 *μ*M compared with untreated differentiated control. Body weight and abdominal fat weight were lowered in spontaneously hypertensive rats (SHRs) administered with buspirone of 10 mg/kg/day for 4 weeks than 8-week untreated group. Triglyceride (TG) level was decreased in SHRs administered with buspirone of 5 and 10 mg/kg/day compared to 8-week untreated group. High-density lipoprotein (HDL)-cholesterol concentration was elevated by buspirone 10 mg/kg/day treatment compared to 8-week untreated group. Blood pressures in SHRs were lowered by buspirone treatments of 5 and 10 mg/kg/day compared with 8-week untreated group. Protein levels for peroxisome proliferator-activated receptor *δ* (PPAR*δ*), 5′ adenosine monophosphate-activated protein kinase (AMPK), and PPAR*γ* coactivator-1 alpha (PGC-1*α*) were increased both in C_2_C_12_ cells treated by buspirone of 100 *μ*M and in SHRs administered by buspirone of 1, 5, and 10 mg/kg/day compared to untreated control cells and 8-week untreated group. Fat cell numbers decreased in 8-week untreated group were increased in SHRs administered by buspirone treats of 1, 5, and 10 mg/kg/day. Protein expression levels for angiotensin II type 1 receptor (AT1R) and vascular cell adhesion molecule 1 (VCAM1) were increased in 8-week untreated group compared to 4-week group, however, they were decreased by buspirone treatments of 1, 5, and 10 mg/kg/day.

**Conclusion:**

Buspirone may induce the losses of body weight and abdominal fat weight through the activation of PPAR*δ* dependent catabolic metabolism producing energy, and eventually, the ameliorated lipid metabolism could normalize high blood pressure.

## 1. Introduction

The prevalence rate of obesity is rapidly increasing globally [1], and it is intimately correlated with other diseases such as type 2 diabetes, non-alcoholic fatty liver disease, and hypertension. In addition, obesity exhibits the accumulation of abdominal fat generally and induces dyslipidemia. Adipose tissue augmented by obesity secretes inflammatory cytokines such as tumor necrosis factor-*α* (TNF-*α*), interleukin-1 (IL-1), interleukin-6 (IL-6), and monocyte chemoattractant protein-1 (MCP-1), which aggravate obesity-related metabolic diseases, such as dyslipidemia, insulin resistance, atherosclerosis, and hypertension [2]. In particular, hypertension is the main inducer of cardiovascular diseases [3], so obesity-induced hypertension can be a very dangerous factor to individual health. Thus, it is important and urgent to develop a more effective and safe medicine to cure both obesity and hypertension.

Serotonin (5-hydroxytryptamine [5-HT]) participates in various physiological processes such as digestion and metabolic homeostasis as well as mood modulation [4]. Moreover, serotonin induces body weight loss via appetite suppression [5] and improves high-fat diet-induced obesity through the activation of energy expenditure metabolism [6]. In another research, serotonin administration lowered blood pressure in spontaneously hypertensive rats (SHRs) [7]. Based on reports of anti-obesity and blood pressure modulation of serotonin, and because buspirone acts as a partial agonist for the serotonin 5-HT1A receptor leading to its clinical use as an anxiolytic drug, we can postulate the anti-obesity and anti-hypertensive effects of buspirone, which functions as an agonist for serotonin receptor. The anti-hypertensive effect of buspirone has been reported previously. Namely, chronic administration of buspirone of 1 mg/kg/day decreased blood pressure in a hypertensive rat model, without affecting blood pressure in normotensive rats [8]. However, the molecular biological mechanism for the anti-hypertensive effect of buspirone has not been elucidated. Therefore, this study was conducted to elucidate the precise mechanism for the anti-obesity and anti-hypertensive effect of buspirone administered as the concentrations of 1, 5, and 10 mg/kg/day in SHRs as an obese, hypertensive rat model.

## 2. Methods

### 2.1. Materials

3T3-L1 mouse embryonic fibroblasts and C_2_C_12_ mouse myoblasts were purchased from Korean Cell Line Bank (Seoul, Korea). Reagents for cell culture such as media, fetal bovine serum (FBS), and antibiotic–antimycotic solution (AA) were supplied from WELGENE, Inc. (Daegu, Korea). PRO-PREP™ protein extraction solution and pre-stained protein marker were bought from Intron Biotechnology (Seongnam-si, Gyeonggi-do, Korea). Hematoxylin and eosin (H&E), Oil Red O, sodium succinate, nitroblue tetrazolium (NBT), potassium phosphate monobasic, sodium phosphate dibasic, and protease inhibitor cocktail were supplied from Sigma-Aldrich (St. Louis, MO, USA). A Clarity™ Western ECL Substrate Kit was obtained from Bio-Rad (Hercules, CA, USA). X-ray film was bought from Agfa (Mortsel, Belgium, Germany), and the developer and fixer reagents were purchased from Carestream Health, Inc. (Rochester, NY, USA). Buspirone was purchased from Boryung Pharmaceutical Co., Ltd. (Seoul, Korea). SHRs were purchased from Doo-Yeol Biotech (Seoul, Korea), while the animal diets were purchased from Central Lab. Animal Inc. (Seoul, Korea). Primary antibodies against 5′ adenosine monophosphate kinase (AMPK), phosphorylated AMPK (p-AMPK), and endothelial nitric oxide synthase (eNOS) were bought from Cell Signaling Technology, Inc. (Danvers, MA, USA). Primary antibodies for peroxisome proliferator-activated receptor delta (PPAR*δ*) and peroxisome proliferator-activated receptor gamma coactivator-1 alpha (PGC-1*α*) were supplied from Abcam (Cambridge, UK). Primary antibodies for vascular cell adhesion molecule 1 (VCAM1) and secondary antibodies were purchased from Santa Cruz Biotechnology, Inc. (Dallas, TX, USA). Primary antibody for angiotensin II type 1 receptor (AT1R) was supplied from Abcam (Cambridge, UK). VECTASTAIN® Elite® ABC-HRP Kit and DAB peroxidase substrate kit were bought from Vector Laboratories, Inc. (Newark, CA, USA). Reagents to quantify the concentrations of total cholesterol and high-density lipoprotein cholesterol (HDL cholesterol) were bought from Kyowa Medex Co., Ltd. (Tokyo, Japan). Reagent to estimate the level of triglyceride (TG) was supplied from Abcam (Cambridge, UK).

### 2.2. Cell Culture

C_2_C_12_ myoblasts were cultured in Dulbecco's Modified Eagle's Medium (DMEM) including 10% FBS and 1% AA solution in 37°C, 5% CO_2_ incubator. The medium was replaced with fresh one every 48–72 hours. When the C_2_C_12_ myoblasts reached to 80% confluence, they were placed in differentiation medium containing 1% FBS and 1% AA and differentiated to myotubes for 72 hours. Buspirone of 100 *μ*M, as a final concentration, was treated to the cells at differentiation stage for 48 hours and incubated for 24 hours. 3T3-L1 preadipocytes were cultured in DMEM containing 10% calf serum and 1% AA solution at 37°C in a 5% CO_2_ incubator. The medium was replaced every 48–72 hours. 3T3-L1 cells between 8 and 17 passages were plated at a density of 5 × 10^4^ cells per well in 24-well culture dishes in DMEM containing 10% calf serum and 1% AA solution. When 3T3-L1 cells reached confluence, the existed medium was replaced with differentiation medium. The differentiation medium contained 0.0125 *μ*mol/mL dexamethasone, 12.5 *μ*mol/mL 3-isobutyl-1-methylxanthine, 10 *μ*g/mL insulin, and 10% FBS. Buspirone of 50 *μ*M and 100 *μ*M, as a final concentration, was added to the differentiation medium. After differentiation for 2 days, the medium was replaced with insulin medium, which included 10 *μ*g/mL insulin and 10% FBS. After incubation in insulin media for 2–4 days, the medium was replaced with maintenance medium, which contained only 10% FBS. Buspirone is added to the differentiation medium step.

### 2.3. Animal Experiment

Three-week-old male SHRs were bred in standardized conditions (21°C, 41–62% humidity) with a regular day/night (10/14 hours) cycle and free access to water and a laboratory diet. After 1 week of acclimation, the assigned rats were divided into five groups: 4w (basal normotensive control; sacrificed at 4 weeks of age), 8w (untreated disease control; sacrificed at 8 weeks of age), buspirone 1 mg/kg/day, buspirone 5 mg/kg/day, and buspirone 10 mg/kg/day. The occurrence of hypertension in SHRs has been known to begin at the 6 weeks age [9, 10]. However, SHRs of 4 weeks age were preferred to determine the early (4-week) anti-hypertensive effects of buspirone in our study.

Buspirone was administered to SHRs for 4 weeks starting at 4 weeks of age. Buspirone was administered to SHRs via drinking water in a 500 mL bottle, and the method to make drinking water containing buspirone is as follows: we measured mean body weight per each experimental group once a week during whole experimental period, and the weight of buspirone to be dissolved was calculated based on mean body weight per each group and average daily water intake amount per rat. An average consumption amount of drinking water per rat body weight 100 g has been known 10 mL/day [11]. Thus, the concentrations of buspirone 1, 5, and 10 mg/kg/day can be converted to 0.01, 0.05, and 0.1 mg/mL/kg/day in each.

This animal experiment started at 4 weeks of age to elucidate the effects and the action mechanism of buspirone on the hypertension of early stage. Blood pressure was measured with tail cuff plethysmography every week using a BP-2000 Blood Pressure Analysis System™ in all SHRs (Visitech Systems Inc.; Apex, NC, USA).

Animals in group 4w were euthanized at 4 weeks of age, whereas rats in the other groups were euthanized after 4 weeks of treatment at 8 weeks of age. Blood samples were acquired from the inferior vena cava, and skeletal muscle and visceral fat tissues were dissected properly and weighed. Harvested organs were stored in a −80°C refrigerator or in 10% (w/v) formalin for immersion fixation. All animal experiments were in accordance with the Animal Experiment Ethics Guide of Guro Hospital, Korea University. All animal experiments were accomplished according to the Korea University Animal Science Rules and Regulations, and the protocols were approved by the Korea University Institutional Animal Care and Use Committee (approval number: KUIACUC-2011-176).

### 2.4. Western Blotting

Cells and tissues were homogenized with protein extracting solution, and protein amounts of cell and tissue extracts were measured by the Bradford method. The extracted proteins of 10 *μ*g were loaded onto 10% sodium dodecyl sulfate–polyacrylamide gel electrophoresis (SDS–PAGE) gels. Protein blotting to nitrocellulose membranes was done for 90 minutes at 100 volts, and the membranes were blocked overnight in 5% skimmed milk solution and washed three times for 10 minutes with Tris-buffered saline containing 0.05% tween 20 (TBS-T). The membranes were incubated with primary antibodies at room temperature for 2 hours. Dilution conditions for primary antibodies were as follows: PPAR*δ*, AMPK, p-AMPK (at Thr172), and PGC-1*α* were 1 : 1000. After the additional washing three times for 10 minutes, with TBS-T, the membranes were incubated with horseradish peroxidase conjugated secondary antibodies at room temperature for 1 hour. Dilution conditions for secondary antibodies were as follows: anti-rabbit IgG antibodies for PPAR*δ*, AMPK, p-AMPK, and PGC-1*α* were 1 : 10,000. Afterward, the membranes were washed three times for 10 minutes with TBS-T and once with TBS for 10 minutes, and the membranes were treated with chemiluminescent substrate and enhancer solutions. Images were acquired manually using developer and fixer reagents, and the results were analyzed by ImageJ program.

### 2.5. H&E Staining

Visceral fat tissues, isolated from all SHRs, were harvested and fixed in 4% paraformaldehyde. The samples were embedded in paraffin and cut into slices (4–5 *μ*m thick) using microtome, and the slices were stained with H&E. They were further visualized using an optical microscope (Olympus BX51, Tokyo, Japan) and photographed.

### 2.6. Immunohistochemistry

Aorta tissue slides were manufactured from frozen sections, and then the tissues on the slides were fixed with 4% paraformaldehyde solution for 20 minutes. The slides were treated with 0.3% H_2_O_2_ solution for 10 minutes to get rid of endogenous peroxidase activity, and after washing, they were blocked with normal serum solution for 1 hour. The slides were reacted with primary antibodies for AT1R and VCAM1 (the dilution rate of 1 : 100) for 1 hour and washed with phosphate buffered saline (PBS). Secondary antibody was added to slides and reacted for 30 minutes. After washing them with PBS, the slides were reacted with premixed VECTASTAIN ABC reagent solution for 30 minutes. After PBS washing, the slides were reacted with DAB substrate solution until color appeared. The slides were washed by tab water, dried, and mounted. Observation and photograph acquisition were done by optical microscope (Olympus BX51, Tokyo, Japan).

### 2.7. Succinate Dehydrogenase (SDH) Activity Assay

Succinate dehydrogenase (SDH) activity assay is a modified method of previously reported protocol [12]. Skeletal muscle tissue samples were homogenized in PBS containing a 1% (v/v) protease inhibitor cocktail solution. The homogenized tissue extracts were centrifuged at 13,000 rpm and 4°C for 5 minutes, and then, the supernatants were transferred to new tubes; the supernatants were used as enzyme solutions. The incubation solution consisted of 1 M phosphate buffer (25 *μ*L), 0.2 M sodium succinate (125 *μ*L), 10 mg/mL NBT (25 *μ*L), and distilled water (235 *μ*L) per reaction, and samples were incubated for 20 minutes at a 37°C. Enzyme solution (90 *μ*L) was added to the prewarmed incubation solution (410 *μ*L), and the reaction mixture was incubated for 30 minutes at a 37°C. After the reaction is over, the absorbance of the reaction mixture was measured at 550 nm. Enzyme activity was calculated using the following formula:

(1) $$\begin{array}{c} \text{Enzyme activity}=(\text{absorbance of the enzyme reaction}-\text{absorbance of the diluted enzyme solution})/\text{quantified protein amount}. \end{array}$$

### 2.8. Statistics

Experimental results are expressed as mean ± SEM (standard error of mean). Statistically significant differences between two groups were calculated by the unpaired *t*-test, and to compare means of three or more groups was used one-way ANOVA test and followed by Bonferroni's multiple comparisons test. The *p* value of <0.05 was considered significant.

## 3. Results

### 3.1. Effect of Buspirone on Lipid Accumulation in Differentiated 3T3-L1 Cells

**Figure 1:**
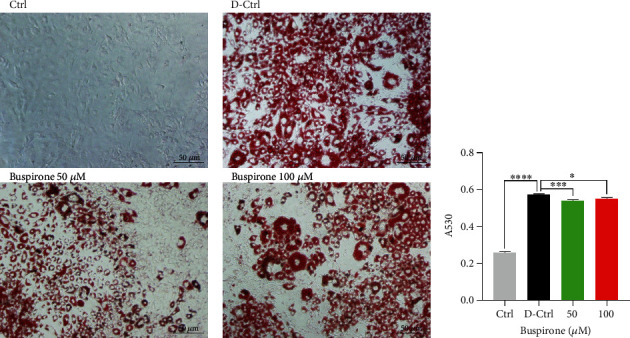
Oil Red O staining of differentiated 3T3-L1 cells treated with buspirone 50 *μ*M or 100 *μ*M. Lipid accumulation (a) and eluted Oil Red O dye content (b) in differentiated 3T3-L1 cells were significantly lowered by the treatments of buspirone. The results are expressed as mean ± standard error of the mean (*n* = 6 ~ 8). Values were statistically analyzed using the unpaired *t*-test to compare two groups, and to compare means of three or more groups was used one-way ANOVA test and followed by Bonferroni's multiple comparisons test. All experiments were repeated over three times. ∗∗*p* < 0.01, ∗∗∗*p* < 0.001, ∗∗∗∗*p* < 0.0001.

Accumulated lipid amount in differentiated 3T3-L1 cells was decreased by the treatments of buspirone of both 50 and 100 *μ*M compared to the untreated differentiated cells (Figure 1). But there was no statistical difference between 50 and 100 *μ*M treated groups.

### 3.2. Effects of Buspirone on Protein Expression Levels for PPAR*δ*, p-AMPK, and PGC-1*α* in C_2_C_12_ Myotubes Treated with Buspirone

**Figure 2:**
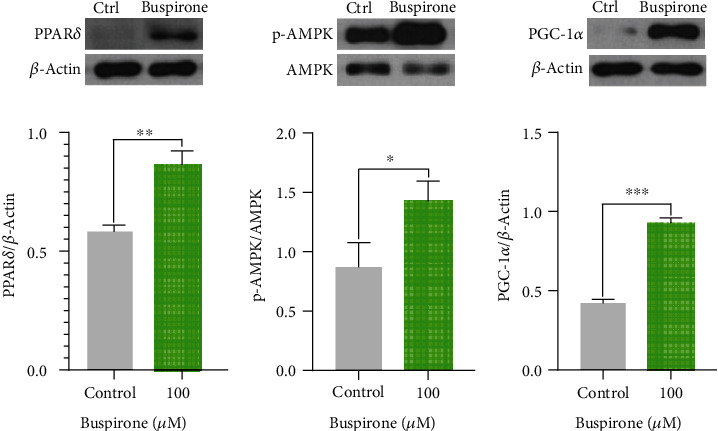
Western blot analyses for PPAR*δ*, p-AMPK, and PGC-1*α* in C_2_C_12_ cells treated with buspirone 100 *μ*M. Buspirone increased the protein levels for PPAR*δ* (a), p-AMPK (b), and PGC-1*α* (c) compared to untreated control in C_2_C_12_ myotubes. The results are expressed as mean ± standard error of the mean (*n* = 3). Values were statistically analyzed using the unpaired *t*-test to compare two groups. All experiments were repeated over three times. ∗*p* < 0.05, ∗∗*p* < 0.01, ∗∗∗*p* < 0.001.

Buspirone elevated protein levels for PPAR*δ*, p-AMPK, and PGC-1*α* compared to control group in C_2_C_12_ myotubes treated with buspirone 100 *μ*M for 48 hours (Figure 2). These results suggest that buspirone can sequentially regulate the metabolic pathway of PPAR*δ*, p-AMPK, and PGC-1*α*.

### 3.3. Effects of buspirone on the weights of body and abdominal fat, the levels of triglyceride and blood pressure, and the HDL-cholesterol level in SHRs

**Figure 3:**
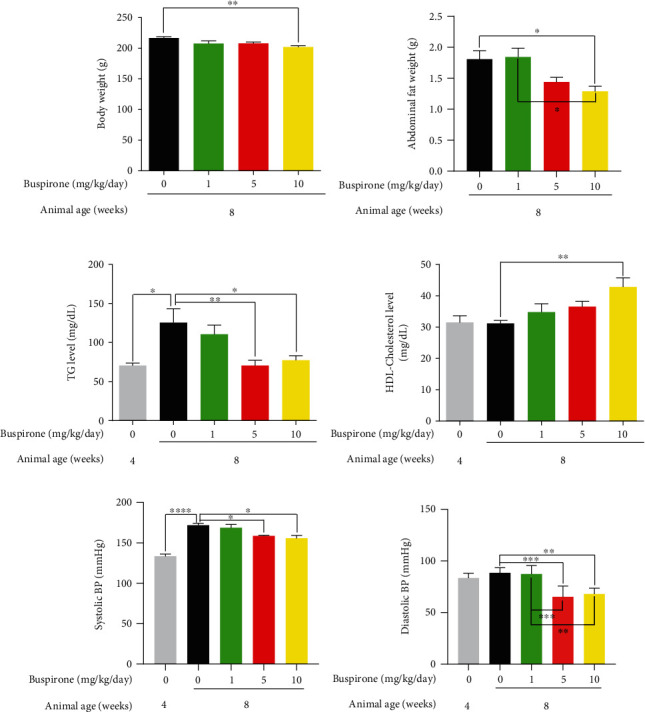
Body weight, abdominal fat weight, triglyceride and HDL-cholesterol concentrations, and systolic and diastolic blood pressure levels in the serum of SHRs treated with buspirone 1, 5, or 10 mg/kg/day (0.01, 0.05, or 0.1 mg/mL/kg/day). Body weight and abdominal fat weight were decreased by the treatment buspirone 10 mg/kg/day (0.1 mg/mL/kg/day) compared to 8w untreated group (a and b). Triglyceride concentration was decreased in the groups treated with buspirone 5 and 10 mg/kg/day (0.05 and 0.1 mg/mL/kg/day) (c). HDL-cholesterol concentration was more elevated by buspirone 10 mg/kg/day (0.1 mg/mL/kg/day) than 8w (d). Systolic blood pressure increased at 8 weeks age SHRs was decreased in buspirone 5 and 10 mg/kg/day (0.05 and 0.1 mg/mL/kg/day) treated groups (e), and diastolic blood pressure level was lowered in buspirone 5- or 10 mg/kg/day (0.05- and 0.1 mg/ml/kg/day) treated group compared to 8w (f). The results are expressed as mean ± standard error of the mean (*n* = 5 ~ 7). Values were statistically analyzed using the unpaired *t*-test to compare two groups, and to compare means of three or more groups was used one-way ANOVA test and followed by Bonferroni's multiple comparisons test. All experiments were repeated over three times. ∗*p* < 0.05, ∗∗*p* < 0.01, ∗∗∗*p* < 0.001.

The body weight and abdominal fat weight were decreased in SHRs treated with buspirone of 10 mg (0.1 mg/mL) compared to the 8w untreated group (Figures 3(a) and 3(b)). In abdominal fat weight result, a statistically significant difference was between 1 mg buspirone treated group and 10 mg buspirone treated group.

The systolic and diastolic blood pressure levels and the TG concentration were decreased by the buspirone treat of 5 and 10 mg (0.05 and 0.1 mg/mL) compared to the untreated 8w (Figures 3(c), 3(e), and 3(f)). The diastolic blood pressure of groups treated with buspirone of 5 and 10 mg (0.05 and 0.1 mg/mL) was significantly lowered than buspirone 1 mg treated group. The HDL-cholesterol concentration was more increased in 10 mg (0.1 mg/mL) buspirone treated group than the 8w untreated group (Figure 3(d)). From these results, we confirmed that the amelioration effects of buspirone for weight loss and dyslipidemia were the most in 10 mg (0.1 mg/mL) buspirone treated group.

### 3.4. Effect of Buspirone on Adipocyte Numbers in Abdominal Fat from SHRs

**Figure 4:**
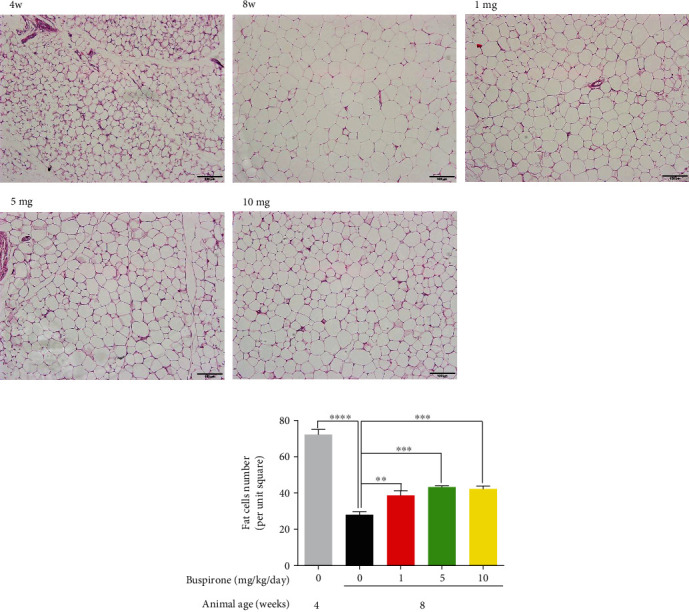
Estimation of fat cell number in abdominal fat tissues from SHRs treated with buspirone 1, 5, or 10 mg/kg/day (0.01, 0.05, or 0.1 mg/mL/kg/day). Abdominal fat tissue slides of SHRs were fixed by formaldehyde and stained with hematoxylin and eosin dyes (a). Magnification is 100 folds. The number of fat cells per unit square was the largest in 4w, and the number was the least in 8w. The decreased fat cell number was increased by buspirone treatments (b). The results are expressed as mean ± standard error of the mean (*n* = 5). Values were statistically analyzed using the unpaired *t*-test to compare two groups, and to compare means of three or more groups was used one-way ANOVA test and followed by Bonferroni's multiple comparisons test. All experiments were repeated over three times. ∗∗*p* < 0.01, ∗∗∗*p* < 0.001, ∗∗∗∗*p* < 0.0001.

When the numbers of adipocytes per unit square (35.88 *μ*m^2^) were measured in abdominal fat tissue slides stained with H&E reagents, the adipocytes numbers in the 8w untreated group decreased to less than 50% of that in the 4w group. However, the adipocytes numbers increased in SHRs treated with buspirone 1, 5, and 10 mg/kg/day (0.01, 0.05, and 0.1 mg/mL/kg/day) than in the 8w untreated SHRs (Figure 4). But there were no statistical differences among buspirone 1, 5, and 10 mg/kg/day (0.01, 0.05, and 0.1 mg/mL/kg/day) treated groups.

### 3.5. Effects of Buspirone on the Protein Levels for PPAR*δ*, p-AMPK, and PGC-1*α* in Abdominal Fat Tissues from SHRs

**Figure 5:**
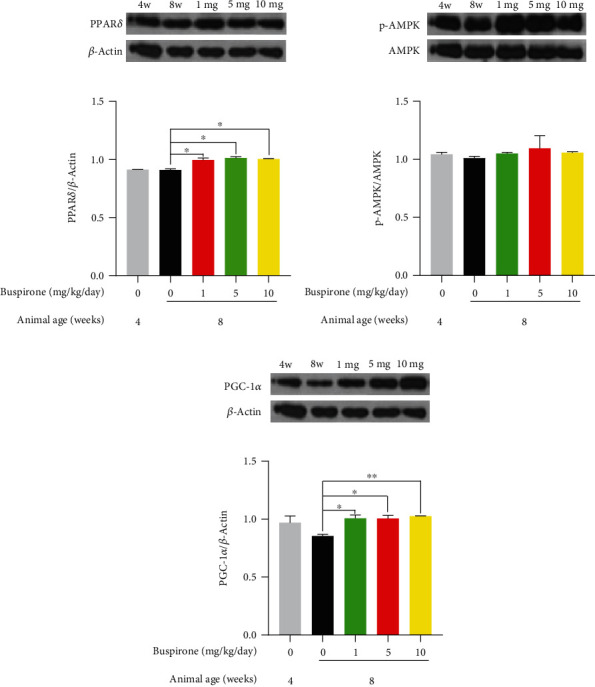
Western blot analyses for PPAR*δ*, p-AMPK, and PGC-1*α* in the abdominal fat tissues of SHRs treated with buspirone of 1, 5, or 10 mg/kg/day (0.01, 0.05, or 0.1 mg/mL/kg/day). PPAR*δ* expression was increased in groups treated with buspirone of 1, 5, and 10 mg/kg/day (0.01, 0.05, and 0.1 mg/mL/kg/day) compared to 8w untreated group (a). Phosphorylated AMPK level was not significantly elevated in groups treated with buspirone 1, 5, and 10 mg/kg/day (0.01, 0.05, and 0.1 mg/mL/kg/day) (b). PGC-1*α* protein expression was increased in groups treated with buspirone of 1, 5, and 10 mg/kg/day (0.01, 0.05, and 0.1 mg/ml/kg/day) (c). The results are expressed as mean ± standard error of the mean (*n* = 2 ~ 3). Values were statistically analyzed using the unpaired *t*-test to compare two groups, and to compare means of three or more groups was used one-way ANOVA test and followed by Bonferroni's multiple comparisons test. All experiments were repeated over three times. ∗*p* < 0.05, ∗∗*p* < 0.01.

The PPAR*δ* protein level was increased by the buspirone treatments of 1, 5, and 10 mg/kg/day (0.01, 0.05, and 0.1 mg/mL/kg/day) compared to the 8w untreated group; however, there were no differences among buspirone 1, 5, and 10 mg/kg/day (0.01, 0.05, and 0.1 mg/mL/kg/day) treated groups (Figure 5(a)). The p-AMPK protein expression levels were not statistical significances in buspirone treated groups compared with 8w untreated group. This result might be caused by the high individual differences among SHRs (Figure 5(b)). The protein level for PGC-1*α* was elevated in groups treated by buspirone of 1, 5, and 10 mg/kg/day (0.01, 0.05, and 0.1 mg/mL/kg/day) compared to the 8w untreated group; however, there were no differences among buspirone 1, 5, and 10 mg/kg/day (0.01, 0.05, and 0.1 mg/mL/kg/day) treated groups (Figure 5(c)). These results suggest that buspirone treatments of three concentrations almost equally affected the expression levels for PPAR*δ*, AMPK, and PGC-1*α*.

### 3.6. Effects of Buspirone on SDH Activity and Protein Expression for PPAR*δ*, p-AMPK, and PGC-1*α* in Skeletal Muscle Tissues from SHRs

**Figure 6:**
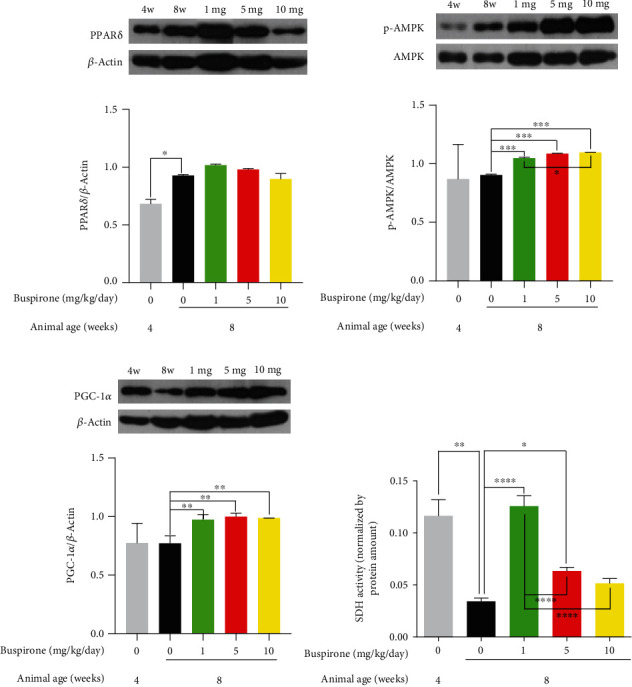
SDH activity assay and western blot analyses for PPAR*δ*, p-AMPK, and PGC-1*α* in skeletal muscle tissues from SHR treated with buspirone 1, 5, or 10 mg/kg/day (0.01, 0.05, or 0.1 mg/mL/kg/day). PPAR*δ* expression was not significantly increased in groups treated with buspirone 1, 5, and 10 mg/kg/day (0.01, 0.05, and 0.1 mg/mL/kg/day) compared to 8w untreated group (a). Phosphorylated AMPK and PGC-1*α* levels were elevated in groups treated with buspirone 1, 5, and 10 mg/kg/day (0.01, 0.05, and 0.1 mg/mL/kg/day) (b and c). SDH activity was lowered in 8w untreated group compared to 4w normal group; however, the activity was increased in groups treated with buspirone 1, 5, and 10 mg/kg/day (0.01, 0.05, and 0.1 mg/mL/kg/day) (d). The results are expressed as mean ± standard error of the mean (*n* = 3 ~ 5). Values were statistically analyzed using the unpaired *t*-test to compare two groups, and to compare means of three or more groups was used one-way ANOVA test and followed by Bonferroni's multiple comparisons test. All experiments were repeated over three times. ∗*p* < 0.05, ∗∗*p* < 0.01, ∗∗∗*p* < 0.001, ∗∗∗∗*p* < 0.0001.

The protein level for PPAR*δ* merely showed increased trends in the buspirone treated groups compared to the 8w untreated group. These results might be caused by the high individual differences in SHRs (Figure 6(a)). The p-AMPK expression was elevated in groups treated with buspirone of 1, 5, and 10 mg/kg/day (0.01, 0.05, and 0.1 mg/mL/kg/day) compared to the control, and the statistical significance between buspirone 1 and 10 mg/kg/day (0.01 and 0.1 mg/mL/kg/day) treated groups was confirmed (Figure 6(b)). The PGC-1*α* protein level was increased in groups treated with buspirone of 1, 5, and 10 mg/kg/day (0.01, 0.05, and 0.1 mg/mL/kg/day); however, there were no differences among buspirone 1, 5, and 10 mg/kg/day (0.01, 0.05, and 0.1 mg/mL/kg/day) treated groups (Figure 6(c)). SDH activity in the 8w group was decreased compared to the 4w group; however, the decreased activity was increased in skeletal muscle tissues from groups treated with buspirone of 1, 5, and 10 mg/kg/day (0.01, 0.05, and 0.1 mg/mL/kg/day) (Figure 6(d)). The SDH activity in skeletal muscle tissues of SHRs was the highest in the group treated by buspirone of 1 mg; it is supposed that the SDH activity was the most sensible to the buspirone concentration of 1 mg/kg/day.

### 3.7. Effects of Buspirone on the Protein Expressions for AT1R and VCAM1 in Aorta from SHRs

**Figure 7:**
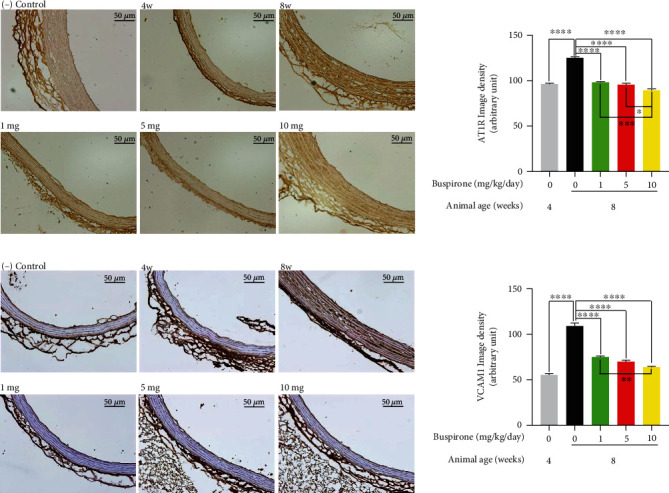
Immunohistochemistry for angiotensin II type 1 receptor (AT1R) and vascular cell adhesion molecule 1 (VCAM1) in aorta slides from SHRs. Expressions for AT1R and VCAM1 were elevated in 8w untreated group compared to 4w normal group; however, the increased expressions were decreased in all buspirone treated groups (a and b). The results are expressed as mean ± standard error of the mean (*n* = 10). Values were statistically analyzed using the unpaired *t*-test to compare two groups, and to compare means of three or more groups was used one-way ANOVA test and followed by Bonferroni's multiple comparisons test. All experiments were repeated over three times. ∗∗∗∗*p* < 0.0001.

The protein expressions for AT1R and VCAM1 were elevated in aorta from the hypertensive 8w SHRs compared to normo-tensive 4w SHRs; however, the increased protein levels reverted to normal state by buspirone treatments of 1, 5, and 10 mg/kg/day (0.01, 0.05, and 0.1 mg/mL/kg/day) (Figure 7). In particular, the expressions of AT1R and VCAM1 in aorta from 8w SHRs were the lowest in buspirone treated group of 10 mg/kg/day (0.1 mg/mL/kg/day).

## 4. Discussion

Many clinical and *in vivo* studies reported that blood pressure level is proportional to obesity or weight gain grade [13–15], moreover, the positive association between visceral fat percentage and hypertension was also reported in several researches [16–19].

In addition, increased visceral fat mass induces an increase in levels of cholesterol and TGs [17, 20], and the excessive visceral fat accumulation induces abnormal vascular states, such as vascular stiffness, endothelial dysfunction, and atherosclerosis via inflammation and oxidative stress; eventually, the abnormal vascular state progresses to hypertension [21]. Thus, obesity and hypertension may have a very intimate commonality of pathophysiology; therefore, an integrated study of these two diseases is necessary, whereby the development of an effective and safe drug for both obesity and hypertension can be facilitated.

Although buspirone has been clinically prescribed to cure anxiety disorders, it lowered the blood pressure of healthy volunteers in clinical trial [22], also significantly decreased blood pressure in deoxycorticosterone acetate (DOCA)-salt-induced hypertensive rats [8]. In the case of obesity or weight gain, some reports described not a significant correlation between obesity and buspirone [23, 24]. But in one study, buspirone administration-induced weight loss in streptozotocin-induced diabetic rats fed a high-fat diet [25]. In our study, buspirone ameliorated blood pressure, lipid and cholesterol profiles, body weight, and abdominal fat weight and its effects may be dependent to common metabolic biomarkers. Moreover, the effect of buspirone on the decrease of abdominal fat weight is additionally proved by that buspirone increased the adipocytes numbers in abdominal fat tissue. Because the inverse interrelation between the number and size of fat cells has been established, so counting adipocytes number per unit area can reflect adipogenicity or lipid accumulation [26].

Generally, the elevation of energy consumption rate is an important factor in obesity and hypertension, and PPAR*δ*, AMPK, and PGC-1*α* are involved in catabolic metabolism producing ATP.

PPAR*δ* is an essential modulator for lipid biosynthesis and oxidative phosphorylation; PPAR*δ* gene transduction lowered fat accumulation in adipose and liver tissues and body weight of obese animals, and its activation by an agonist also decreased lipid contents in fat and liver and increased fatty acid oxidation in adipose tissue [27]. In special, the anti-obesity and weight loss effects of telmisartan (AT1R blocker) are dependent to PPAR*δ* [28], also fimasartan, a novel AT1R blocker, ameliorates nonalcoholic fatty liver disease through PPAR*δ* activation [26]. Furthermore, PPAR*δ* is involved in the restoration of endothelial dysfunction, and vasodilation in animals fed a high-fat diet [29]. In addition, PPAR*δ* functions as an upper regulator for AMPK in various cell types [30, 31].

AMPK senses the energy state (ATP:ADP ratio) in cells and then maintains an energy balance via the modulation of ATP production and consumption—namely, AMPK functions as a key regulator for energy homeostasis [32]. AMPK activation by a synthetic chemical increased fatty acid oxidation in liver, skeletal muscle, and adipose tissue from diabetic animals [33]. Moreover, AMPK stimulation by a natural compound improved hyperlipidemia and fatty liver and lowered the body weight in obese animals [34].

In addition, the administration of metformin, an AMPK activator, reduced pulmonary artery pressure in a pulmonary hypertension animal model [35], and AMPK directly regulates PGC-1*α* via phosphorylation [36]. PGC-1*α* is a coactivator that participates in various physiological processes, such as mitochondrial biogenesis, thermogenesis, and fatty acid metabolism, and is highly expressed in brown adipose tissue, heart, and skeletal muscle, wherein mitochondrial numbers and energy-producing catabolic metabolism are elevated [37]. So, PGC-1*α* affects lipid metabolism and metabolic diseases as follows. PGC-1*α* overexpression increases fatty acid oxidation in liver tissue and skeletal muscle cells; however, PGC-1*α* decreases the triacylglycerol content in hepatic tissue [38, 39]. In addition, PGC-1*α* does an important role in an obesity-induced disease; thus, obesity downregulates PGC-1*α* expression, and the diminished PGC-1*α* level decreases the expression of mitochondrial genes and, finally, insulin resistance is induced [40]. Moreover, PGC-1*α* overexpression ameliorates angiotensin II-induced hypertension via the regulation of eNOS [41]. SDH is one of the molecules that are stimulated by PGC-1*α* and is an essential component in both the citric acid cycle and the electron transport system in mitochondria, and the elevations of the mitochondrial fraction and oxidative phosphorylation in skeletal muscle are intimately correlated with weight loss [42, 43]. In this study, buspirone increased the protein levels of PPAR*δ*, AMPK, and PGC-1*α* in skeletal muscle and abdominal fat tissues from SHRs and elevated the SDH activity in the skeletal muscle. Thus, we can suppose that buspirone activated an energy-producing catabolic procedure in abdominal fat and skeletal muscle tissues, and then the stimulated pathway induced the decrease of fat mass and body weight, and it might contribute to the amelioration of high blood pressure. Moreover, buspirone directly induced the normalization of hypertension through reducing the expressions for AT1R and VCAM1 related to atherosclerosis. The correlation among hypertension, AT1R, and VCAM1 has been reported as follows. Angiotensin II induces vasoconstriction after binding to AT1R, and vasoconstriction-induced hypertension provokes atherosclerosis, which causes cardiovascular diseases [44, 45]. VCAM1 does an essential role in the occurrence of atherosclerosis through mediating the adhesion of immune cells such as monocytes and lymphocytes to vascular endothelium [46]. Moreover, blocking of VCAM1 by a neutralizing antibody exhibits a protective effect against Ang II-induced arterial hypertension [47]. So, the expression levels for AT1R and VCAM1 indicate the degree of hypertension.

## 5. Conclusion

**Figure 8:**
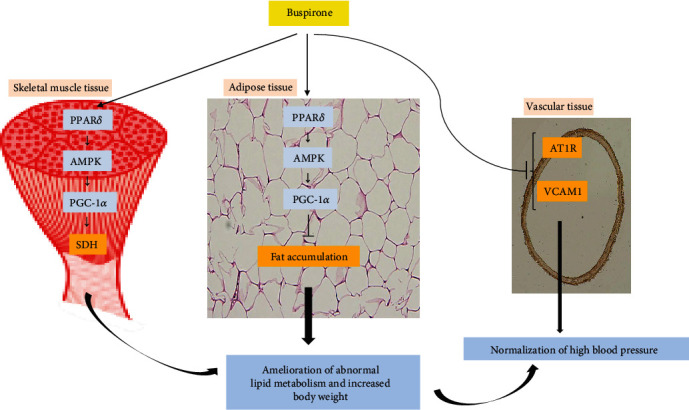
In SHRs, buspirone ameliorates abnormal lipid metabolism and normalizes elevated abdominal fat mass, body weight, and biomarkers related to hypertension. Buspirone ameliorates abnormal lipid metabolism and elevated body weight through stimulating the pathway of PPAR*δ*–AMPK–PGC-1*α* and normalizes blood pressure via the inhibition of AT1R and VCAM1. In addition, the effects of buspirone on skeletal muscle and abdominal fat can affect the modulation of hypertension. Meaning of symbols: arrow indicates activation and up, and horizontal line indicates inhibition.

In conclusion, buspirone may ameliorate the early stage of dyslipidemia, obesity, and hypertension via the pathway of PPAR*δ*–AMPK–PGC-1*α* in hypertensive and obese animals (Figure 8).

In this study, because of feeding normal diet to SHRs, enough obesity did not induce. Thus, in a future study, SHRs should be fed high-fat diet to make more a proper hypertensive and obese rat model, and additional markers such as adiponectin and blood glucose concentration should be estimated. In addition, the more evident lowering effect of buspirone on hypertension shall be proved through a later study using old aged SHRs. Also, the small *N* number in the C_2_C_12_ cell experiments shall be supplemented in a later study.

## Data Availability

We can provide the data when there are requests from other researchers (data available on request). The e-mail addresses for the data request are as follows: mdhsseo@korea.ac.kr, kojung@cau.ac.kr, and lyj2333@hanmail.net.
